# Determinants of climate change awareness level in upper Nyakach Division, Kisumu County, Kenya

**DOI:** 10.1186/s40064-016-2699-y

**Published:** 2016-07-08

**Authors:** Chadwick O. Ajuang, Paul O. Abuom, Esna K. Bosire, Gabriel O. Dida, Douglas N. Anyona

**Affiliations:** School of Environment and Earth Science, Maseno University, P. O. Box 333, 40105 Maseno, Kenya; School of Public Health and Community Development, Maseno University, P. O. Box 333, 40105 Maseno, Kenya

**Keywords:** Climate change markers, Level of awareness, Demographic factors, Upper Nyakach Division, Kenya

## Abstract

Improving the understanding of climate change awareness is one of the top priorities in climate change research. While the African continent is among the regions with the highest vulnerability to climate change, research on climate knowledge and awareness is lacking. Kenya is already grappling with the impacts of climate change, which are projected to increase in a non-linear and non-predictable manner. This study sought to determine climate change awareness levels among households residing in Upper Nyakach Division, Kisumu County, Kenya using common climate change markers viz heavy rainfall, floods, droughts and temperature. A cross-sectional survey design was adopted in which 384 household heads were selected as respondents from 11 sub-locations; all located within Upper Nyakach Division. A questionnaire was used to collect data. Most (90.9 %) respondents had observed changes in the overall climate. Awareness level of climate change varied significantly across the 11 sub-locations. To further gain insight unto which variables were the most significant determinant of climate change awareness in upper Nyakach division, Kisumu county, a Generalized Linear Model (GLM) with Poisson error distribution was built. The model indicated that sex of the household head, education level and age significantly influenced respondents’ awareness to climate change markers. Most (87 %) households reported rising temperatures over the past 20 years. Over half (55.2 %) the respondents had observed declining rains, with significant differences being observed across age groups. Up to 75 % of the respondents reported increased droughts frequency over the last 20 years, with significant differences observed across gender. Most (86.7 %) respondents reported having observed changes in water sources with significant differences reported across age groups. The respondents reported an increased prevalence of malaria with significant differences being observed among the education levels and households’ main livelihoods. The general population of the Upper Nyakach Divison is aware of changing global climate. However, more effort is required in mitigating climate change as per the local settings. Awareness campaign aimed at increasing knowledge of climate change markers among community members is recommended.

**Electronic supplementary material:**

The online version of this article (doi:10.1186/s40064-016-2699-y) contains supplementary material, which is available to authorized users.

## Background

Climate change constitutes one of the twenty-first Century key challenges to development the world over (UNDP 2007). In this respect, climate change and global warming have become issues of global concern in recent decades as clearly evidenced by the flurry of conferences, campaigns, reports and researches on the subject. Despite a few skeptical views (Frank 2008; Lupo 2008; Washington and Cook 2011), there exists widespread consensus among scientists that climate change is happening and is being driven by the unsustainable practices of mankind, especially the burning of fossil fuels, industrial pollution, deforestation, and land use changes (IPCC 2007; Canadel et al. 2011; Weart 2010; Cook et al. 2013). Presently, there is a general consensus that climate change is now a well-established reality (Lindsey et al. 2010*)*.

According to the Inter-governmental Panel on Climate Change, IPCC (2007), observational evidence from all the continents and most oceans shows that many natural systems are already being affected by regional climatic changes. The most obvious manifestation of climate change is the rising temperature, melting of glaciers, rising of sea-level, changes in precipitation patterns, recurrent droughts and devastating floods; all of which are important climate change markers (IPCC 2007). Available scientific evidence for instance shows that the earth experienced an average warming of approximately 0.6 °C during the twentieth Century (IPCC 2001) and is expected to warm by about 2–3 °C by the end of the 21st century (IPCC 2007). According to Holdren (2006), the last 50 years of the twentieth Century were the warmest in 600 years. Several other studies are in concordance that the frequency and severity of droughts and floods which are climate change markers have increased over the past 50 years, especially in the Eastern Africa region (Food and Agriculture Organization Statistics [FAOSTAT] 2000; United Nations Environmental Program [UNEP] 2002).

It is projected that continued green house gas (GHG) emissions would induce many changes in the global climate system during the twenty-first century that would very likely be larger than those observed during the twentieth century (Pachauri and Spreng 2011). In addition, there is increased likelihood that the impacts of climate change will advance in a non-linear and non-predictable manner. Climate change will have both positive and negative impacts, but the adverse effects will be felt much more strongly in developing countries (IPCC 2001). The negative impacts of climate change varies globally; though small-scale farmers particularly in developing countries suffer the most because of their dependence on rain-fed agriculture, limited financial capacity, low adaptive capacity, high dependence on natural resources, inability to detect the occurrence of extreme hydrological and meteorological events, limited infrastructure, illiteracy, lack of skills, low awareness levels and lack of capacity to diversify (Kurukulasuriya and Mendelssohn 2006). As such, the problem and the challenges of climate change are becoming more threatening to sustainable development and the totality of human existence (Adejuwon, 2004).

Africa is one of the most vulnerable continents to the impacts of climate change (IPCC 2007; Densanker 2002). These adverse impacts of climate change have combined with poverty, poor policy and poor institutional frameworks to make the situation even worse (African Ministerial Conference on Environment [AMCEN] 2011). Ranking high among the impacts of climate change in Africa is food insecurity triggered by severely compromised agricultural production, dwindling fish stocks due to ecosystem changes, reduced livestock production due to grassland degradation and deforestation among others. These impacts are particularly exacerbated by climate change markers such as frequent droughts, unpredictable floods and change in rainfall patterns (Collier et al. 2008).

Studies predict anything up to 200 million more food-insecure people by 2050 or an additional 24 million malnourished children over the same period. Most of these studies suggest that the worst impacts will be felt by the poorest people majority of whom are marginalized and live in developing countries. Considering these facts, climate change has the potential to affect development activities in Africa and can jeopardize the achievement of the Sustainable Development Goal (SDG) no. 13, which focuses on enhancing the resilience of climate change as summarized in the recently published IPCC 5th assessment report (IPCC 2014).

The climate change phenomenon is unmistakably intensifying in Kenya at an alarming rate (Government of Kenya 2010a). The country and the greater East African region is already experiencing high temperatures (Herrero et al. 2010). According to SEI (2009), climate change projections indicate increases in mean annual temperature in Kenya of between 1 and 3.5 °C by the year 2050. This warming is likely to lead to depletion of glaciers on Mount Kenya (IPCC 2007), declining water levels in many rivers and the subsequent interruptions in electricity generation (GoK 2007). The impacts of climate change in Kenya have been severe and have often affected some of the key sectors of the economy due to potential threats to coastal zones as a result of sea level rise, health burdens, energy demand, infrastructure, water resources, agriculture and loss of ecosystem services (Kuria 2009; SEI 2009). The most vulnerable to these impacts are poor rural households that depend on the climate-sensitive sectors for survival (Mutimba et al. 2010).

The impacts of climate change and variability portend significant economic cost to the country. SEI (2009) projects a loss at about 3 % of the Kenya’s GDP each year by 2030. This is likely to slow down Kenya’s economic growth, as projected in the Kenya Vision 2030, more so because the economy is heavily dependent on climate-sensitive sectors such as agriculture, tourism, and coastal zones and that the means to cope with the hazards are weak (GoK 2007).

Owing to observational facts, it is imperative to understand the actual dynamics of climate change markers with reference to temperature, drought, floods and heavy rainfall and their impacts at the community and household levels (Deressa et al. 2008). Awareness of climate change and its impacts among households would prepare them to effectively cope or adapt to the impacts. As Mtambanengwe et al. (2012) observed, an assessment of the level of awareness on climate change and variability may contribute towards the formulation of adaptation strategies designed to improve rural livelihoods and reduce vulnerability. Various studies have revealed that climate change awareness and perception varies within and across regions (Pew Research Centre 2006; Pugliese and Ray 2009).

A recent study by Lee et al. (2015) concurs with past studies that climate change awareness and risk perception were unevenly distributed around the world in 2007–2008, with the highest levels of awareness (over 90 %) reported in the developed world, and by contrast, majorities in developing countries from Africa to the Middle East and Asia reported not having heard of climate change. Earlier climate change awareness studies conducted in the developed world revealed that the awareness level were high among respondents (Pew Research Centre 2006; Pugliese and Ray 2009), but still not a priority environmental issue in most of these countries (Leiserowitz 2006; Pew Research Centre 2013). On the contrary, studies conducted on climate change in developing countries reveal that the vast majority of people were unaware of climate change despite their high vulnerability to the impacts of climate change, (Pew Research Centre 2006; Pugliese and Ray 2009; Godfrey et al. 2009).

Studies show that the majority of Kenya’s population is unaware of climate change and is instead concerned about food insecurity resulting from recurrent droughts and floods in the country (GoK 2010b). The Kenya National Environment Management Authority [NEMA] (2005) points out that even though the public is largely unaware of climate change issues, the depth and scale of this lack of awareness needs to be clearly established. Mutimba et al. (2010) while acknowledging this low level of awareness among Kenyans, at the time of the NEMA assessment, pointed out several global conferences, conventions and events which over the years may have raised the level of climate change awareness in Kenya. These include: the 12th Conference of Parties of the United Nations Framework Convention on Climate Change (UNFCCC) held in Kenya, the drought episodes of 2006–2009, their effects and linkage with local environmental issues such as the conservation of the Mau Forest and the participatory approach employed in the development of the National Climate Change Response Strategy (NCCRS).

Despite these efforts, several other researchers have reported that the level of awareness on climate change and its markers in Africa and particularly among Kenyans is considerably low (GoK 2010a; NEMA 2005; Mutimba et al. 2010 and Pelham 2009**).** A consideration of these facts presented uncertainty about the actual level of awareness on climate change upon which the study carved out a knowledge gap. To address this gap, this study sought to establish the level of awareness of climate change markers among households in Upper Nyakach Division and their effects on demographic factors which are presented herein.

Several studies have reported that national, cultural and geographic factors play a key role in shaping individual-level perceptions and awareness of climate change (Leiserowitz 2006; Brechin and Bhandari 2011). Lee et al. (2015) thus emphasized on the importance of identifying the key individual-level predictors of climate change awareness and risk perception for each country separately. In the same study by Lee et al. (2015), it was reported that a significant proportion of nations had different top-ranked predictors. For instance, awareness and perception of local temperature change is the strongest predictor of risk perceptions in many Asian and African countries. Their findings were particularly important because previous research also established that many individuals around the world have accurately detected recent changes in local temperature anomalies (Howe et al. 2013). The current study sought to use climate change markers viz heavy rainfall, floods, drought and temperature which are commonly encountered and easily recognizable climate change markers to establish the community’s awareness of climate change in the Upper Nyakach Division of Kisumu County, Kenya. This knowledge would bridge the gap between policy formulation and building adaptive capacity to climate change.

## Methods

### Study area

This study was conducted in Upper Nyakach Division of Kisumu County in Western Kenya, which covers total land area of 170.9 Km^2^ (https://opendata.go.ke) and lies between longitudes 34°44′E to 35°15′E and latitudes 0°08′S and 0°27′S (GoK 2009). The altitude ranges from 1100 m along the shores of Lake Victoria to 1800 m on the Nyabondo Plateau (Okere and Kodiwo 2012). The area shares climatic conditions of Lake Victoria Basin with a mean annual rainfall of between 600 to 1630 mm and temperature ranging from 18 to 34 °C. The location experiences two distinct extreme weather conditions—droughts and floods mainly ravaging West Nyakach Location. Water scarcity is often acute after short rainy seasons with the peak in January (Okere and Kodiwo 2012). The Upper Nyakach division has a total of eleven sub-locations *i.e*. Ramogi, East Koguta, East and West Kadianga, Gari, Kajimbo, Anding’o Opanga, Upper Kadianga, West Koguta, Lower Kadiang’a and Nyong’ong’a whose climate is generally similar owing to their proximity to each other (GoK 2009). However, on the basis of their location within Upper Nyakach Division, the 11 sub-locations can be broadly classified into three. Nyongonga and Lower Kadianga are located in the plains bordering Lake Victoria on the lower reaches of River Sondu. These two are prone to flooding and their main source of water is the lake. West Koguta, Upper Kadianga, Anding’o Opanga and Kadianga West, are located in a hilly terrain and both experience semi-arid conditions, with springs and wells serving as the major water sources for the inhabitants. Gari, Kajimbo, East Kadianga, East Kuguta and Ramogi sub-locations are located in a hilly terrain with relatively good weather that supports farming activities. The sources of water for the inhabitants include springs, rivers, wells and ponds. Basing on the 2009 Kenya population and housing census, Upper Nyakach Division has a population of 74,252 persons and a population density of about 435 persons per square kilometers (GoK 2010c). The dominant socio-economic activities for majority of households in the area are agriculture and livestock production for subsistence (Okere and Kodiwo 2012).

### Study design

The study adopted a cross-sectional survey design in which data was collected from both primary and secondary sources between November and December 2013. A total of 384 household heads were randomly selected from 16,133 households for the survey. A structured questionnaire was used to collect household data while Focus Group Discussions (FGD) and Key Informant Interviews (KIIs) were used to collect qualitative data on community awareness of climate change with a view to triangulate the household data. A questionnaire on awareness of climate change markers administered on 364 respondents of Upper Nyakach Division, Kisumu County, Kenya is provided as Additional file 1.

### Data analysis

Quantitative data analyses were aided by the Statistical Package for Social Sciences (SPSS) version 20, Statistical Analysis Software (SAS) version 9.4 and excel spreadsheet softwares. The data was first descriptively analyzed to give frequencies and proportions. Since descriptive statistics were not sufficient to determine significant relationships between dependent and independent variables, Chi square tests, were used to determine the association between variables. The relationship between awareness of climate change markers and the background factors was evaluated using GLM technique, whereby the dependent variable was climate change awareness, while independent variables were households’ head age, education level and gender. A respondent was considered aware of the climate change markers when he or she was able to mention at least four out of the seven climate change markers (i.e. declining frequency of rain, increasing frequency of rain, declining severity of temperature, increasing severity temperature, increasing frequency of floods, decreasing frequency of floods and severity of drought). As the awareness of the climate change markers data were in form of counts and demonstrated clear over-dispersion (evaluated graphically and by comparing the residual deviance with residual degree of freedom), GLMs assuming either Poisson or Negative binomial distribution with appropriate link function was used. Graphical methods were equally applied to assess model fit and ensure a linear relationship existed between the predictor and the link transformed outcome. Analyses were conducted in R version 3.0 (R Core Team, 2013). The threshold for statistical significance for this study was set at 95 % confidence level, thus a *P* value of 0.5 or less (P ≤ 0.05) was considered statistically significant. The findings were presented summarily as texts, tables, and graphs. Qualitative data from KIIs and FGDs transcriptions were analyzed thematically. The results were then presented in form of textual expressions and direct quotations.

## Results and discussion

### Socio-economic profile of the respondents

Results reveal that 4.2 % of the respondents were youth (30–35 years), 71.2 % were middle aged (36–60 years) while 24.5 % were in their old age (above 60 years), clearly showing that the majority of respondents were within the middle age bracket (Table 1). The high proportion of middle aged persons in the study could be explained by the fact that this study targeted household heads, most of whom are in their middle age (36–60 years).

**Table 1:** A contingency table denoting all the variables included in the study and their frequencies

Variable	Codes	N	All %	Valid %	Cumulative %
Gender	0: Male	161	48.2	48.8	48.8
	1: Females	169	50.6	51.2	100.0
	Missing	4	1.2		
Age	0: Youth	14	4.2	4.2	4.2
	1: Middle age	234	70.1	70.9	75.2
	2: Old age	82	24.6	24.8	100.0
	Missing	4	1.2		
Education Level	0: Uneducated	14	4.7	4.7	4.7
	1: Primary	153	51.0	51.0	55.7
	2: Secondary	111	37.0	37.0	92.7
	3: College	15	5.0	5.0	97.7
	4: University	7	2.3	2.3	100.0
	Missing	0	0		
Region/sublocation					
	0: Andingo	22	6.7	6.7	6.7
	1: East Kadian	48	14.5	14.5	21.2
	2: East Koguta	37	11.2	11.2	32.4
	3: Gari	14	4.2	4.2	36.6
	4: Kajimbo	44	13.3	13.3	49.9
	5: Lower Kadian	19	5.8	5.8	55.7
	6: Nyongong	18	5.5	5.5	61.2
	7: Ramogi	16	4.8	4.8	66.0
	8: Upper Kadian	25	7.6	7.6	73.6
	9: West Kadian	37	11.2	11.2	84.8
	10: West Koguta	50	15.2	15.2	100.0
	Missing	0	0		
Gender versus awareness					
	0: Male	146	48.7	48.7	48.7
	1: Female	154	51.3	51.3	100.0
	Missing	0	0		
Age versus awareness					
	0: Youth	14	4.7	4.7	4.7
	1: Middle age	208	69.3	69.3	74.0
	2: Old age	78	26.0	26.0	100.0
	Missing	0	0		
Education versus awareness	0: Uneducated	21	7.0	7.0	7.0
	1: Secondary	153	51.0	51.0	58.0
	2: College	111	37.0	37.0	95.0
	3: University	15	5.0	5.0	100.0
	Missing	0	0		
Livelihood vs awareness					
	0: Crop farming	190	63.3	63.3	63.3
	1: Livestock keeping	4	1.3	1.3	64.6
	2: Employed	70	23.3	23.3	87.9
	3: Trade	21	7.0	7.0	94.9
	4: Fishing	14	4.7	4.7	99.6
	5: Brick making	1	0.3	0.3	100.0
	6. Missing	0	0		
Age vs observed temperature changes					
	1. Youth				
	0: Rising temp	10	71.4	71.4	71.4
	1: Declining temp	0	0	0	71.4
	2: No change	4	28.6	28.6	100.0
	Missing	0	0		
	2. Middle age				
	0: Rising temp	189	90.9	90.9	90.9
	1: Declining temp	5	2.4	2.4	93.3
	2: No change	14	6.7	6.7	100.0
	Missing	0	0		
	3. Old age				
	0: Rising temp	66	80.5	80.5	80.5
	1: Declining temp	11	13.4	13.4	93.9
	2: No change	5	6.1	6.1	100.0
	Missing	0	0		
Age versus observed rainfall changes					
	1. Youth				
	0: Declining rains	8	57.1	57.1	57.1
	1: Late rains	1	7.1	7.1	64.2
	2: Rains coming early	0	0	0	64.2
	3: Shorter rain seasons	3	21.4	21.4	85.6
	4: Longer rain seasons	0	0	0	85.6
	5: More rains	0	0	0	85.6
	6: No change	1	7.1	7.1	92.7
	7: Other	1	7.1	7.1	100.0
	Missing	0	0		
	2. Middle age				
	0: Declining rains	116	50.4	50.4	50.4
	1: Late rains	42	18.3	18.3	68.7
	2: Rains coming early	1	0.4	0.4	69.1
	3: Shorter rain seasons	38	16.5	16.5	85.6
	4: Longer rain seasons	21	9.1	9.1	94.7
	5: More rains	4	1.7	1.7	96.4
	6: No change	0	0	0	96.4
	7: Other	8	3.5	3.5	100.0
	Missing	0	0		
	3. Old age				
	0: Declining rains	58	70.7	70.7	70.7
	1: Late rains	13	16.0	16.0	86.7
	2: Rains coming early	0	0	0	86.7
	3: Shorter rain seasons	6	7.3	7.3	94.4
	4: Longer rain seasons	1	1.2	1.2	95.2
	5: More rains	2	2.4	2.4	97.6
	6: No change	0	0	0	97.6
	7: Other	2	2.4	2.4	100.0
	Missing	0	0		
Age versus drought changes					
	1. Youth				
	0: More frequent	12	85.7	85.7	85.7
	1: Less frequent	2	14.3	14.3	100.0
	2: No change	0	0	0	
	Missing	0	0		
	2. Middle age				
	0: More frequent	168	73.0	73.0	73.0
	1: Less frequent	57	24.8	24.8	97.8
	2: No change	5	2.2	2.2	100.0
	Missing	0	0		
	3. Old age				
	0: More frequent	67	81.7	81.7	81.7
	1: Less frequent	15	18.3	18.3	100.0
	2: No change	0	0	0	
	Missing	0	0		
Gender versus observed temperature changes					
	1. Male				
	0: Rising temperature	141	87.6	87.6	87.6
	1: Declining temperature	12	7.5	7.5	95.1
	2: No change	8	4.9	4.9	100.0
	Missing	0			
	2. Female				
	0: Rising temperature	146	86.4	86.4	86.4
	1: Declining temperature	4	2.4	2.4	88.8
	2: No change	19	11.2	11.2	100.0
	Missing	0	0		
Gender versus observed rainfall changes					
	1. Male				
	0: Declining rains	97	60.2	60.2	60.2
	1: Late rains	35	21.7	21.7	81.9
	2: Rains coming early	0	0	0	81.9
	3: Shorter rain seasons	15	9.3	9.3	91.2
	4: Longer rain seasons	0	0	0	91.2
	5: More rains	5	3.1	3.1	94.3
	6: No change	1	0.6	0.6	95.0
	7: Other	8	5.0	5.0	100.0
	Missing	0	0		
	1. Female				
	0: Declining rains	85	50.3	50.3	50.3
	1: Late rains	21	12.4	12.4	62.7
	2: Rains coming early	1	0.6	0.6	63.3
	3: Shorter rain seasons	36	21.3	21.3	84.3
	4: Longer rain seasons	22	13.0	13.0	97.3
	5: More rains	1	0.6	0.6	97.9
	6: No change	0	0	0	97.9
	7: Other	3	1.8	1.8	100.0
	Missing	0	0		
Gender versus observed flood occurrence					
	1. Male				
	Yes	55	34.2	34.2	34.2
	No	106	65.8	65.8	100.0
	Missing	0	0		
	1: Female				
	Yes	69	40.8	41.1	41.1
	No	99	58.6	58.9	100.0
	Missing	1	0.6		
Education versus observed temperature changes					
	1: Uneducated				
	0: Rising temperature	13	92.9	92.9	92.9
	1: Declining temperature	0	0	0	92.9
	2: No change	1	7.1	7.1	100.0
	Missing	0	0		
	2: Secondary				
	0: Rising temperature	143	82.7	82.7	82.7
	1: Declining temperature	15	8.7	8.7	91.4
	2: No change	15	8.7	8.7	100.0
	Missing	0	0		
	3: College				
	0: Rising temperature	113	94.2	94.2	94.2
	1: Declining temperature	1	0.8	0.8	95.0
	2: No change	6	5.0	5.0	100.0
	Missing	0	0		
	4: University				
	0: Rising temperature	6	85.7	85.7	85.7
	1: Declining temperature	0	0	0	85.7
	2: No change	1	14.3	14.3	100.0
	Missing	0	0		
Education versus observed rainfall changes					
	1: Uneducated				
	0: Declining rains	7	50.0	50.0	50.0
	1: Late rains	6	42.9	42.9	92.9
	2: Rains coming early	0	0	0	92.9
	3: Shorter rain seasons	1	7.1	7.1	100.0
	4: Longer rain seasons	0	0	0	
	5: More rains	0	0	0	
	6: No change	0	0	0	
	7: Other	0	0	0	
	Missing	0	0		
	2: Primary				
	0: Declining rains	92	53.2	53.2	53.2
	1: Late rains	24	13.9	13.9	67.1
	2: Rains coming early	1	0.6	0.6	67.7
	3: Shorter rain seasons	25	14.5	14.5	82.2
	4: Longer rain seasons	22	12.7	12.7	94.9
	5: More rains	2	1.2	1.2	96.1
	6: No change	1	0.6	0.6	96.7
	7: Other	6	3.5	3.5	100.0
	Missing	0	0		
	3: Secondary				
	0: Declining rains	76	63.3	63.3	63.3
	1: Late rains	18	15.0	15.0	78.3
	2: Rains coming early	0	0	0	78.3
	3: Shorter rain seasons	17	14.2	14.2	92.5
	4: Longer rain seasons	0	0	0	92.5
	5: More rains	4	3.3	3.3	95.8
	6: No change	0	0	0	95.8
	7: Other	5	4.2	4.2	100.0
	Missing	0	0		
	4: College				
	0: Declining rains	6	37.5	37.5	37.5
	1: Late rains	2	12.5	12.5	50.0
	2: Rains coming early	0	0	0	50.0
	3: Shorter rain seasons	8	50.0	50.0	100.0
	4: Longer rain seasons	0	0	0	
	5: More rains	0	0	0	
	6: No change	0	0	0	
	7: Other	0	0	0	
	Missing	0	0		
	4: University				
	0: Declining rains	1	14.3	14.3	14.3
	1: Late rains	6	85.7	85.7	100.0
	2: Rains coming early	0	0	0	
	3: Shorter rain seasons	0	0	0	
	4: Longer rain seasons	0	0	0	
	5: More rains	0	0	0	0
	6: No change	0	0	0	0
	7: Other	0	0	0	0
	Missing	0	0	0	
Education versus drought changes					
	1: Uneducated				
	0: Increasing severity	8	57.1	57.1	57.1
	1: Decreasing severity	6	42.9	42.9	100.0
	2: No change	0	0	0	
	Missing	0	0		
	1: Primary				
	0: Increasing severity	109	63.0	63.0	63.0
	1: Decreasing severity	58	33.5	33.5	96.5
	2: No change	6	3.5	3.5	100.0
	Missing	0	0		
	2: Secondary				
	0: Increasing severity	96	80.0	80.0	80.0
	1: Decreasing severity	23	19.2	19.2	99.2
	2: No change	1	0.8	0.8	100.0
	Missing	0	0		
	3: College				
	0: Increasing severity	8	50.0	50.0	50.0
	1: Decreasing severity	8	50.0	50.0	100.0
	2: No change	0	0	0	
	Missing	0	0		
	4: University				
	0: Increasing severity	5	71.4	71.4	71.4
	1: Decreasing severity	1	14.3	14.3	85.7
	2: No change	1	14.3	14.3	100.0
	Missing	0	0		
Education versus observed flood occurrence					
	1: Uneducated				
	0: Yes	5	35.7	35.7	35.7
	1: No	9	64.3	64.3	100.0
	Missing	0	0		
	1: Primary				
	0: Yes	57	32.9	32.9	32.9
	1: No	116	67.1	67.1	100.0
	Missing	0	0		
	2: Secondary				
	0: Yes	55	45.8	45.8	45.8
	1: No	65	54.2	54.2	100.0
	Missing	0	0		
	3 : College				
	0: Yes	5	33.3	33.3	33.3
	1: No	10	66.7	66.7	100.0
	Missing	0	0		
	4: University				
	0: Yes	2	28.6	28.6	28.6
	1: No	5	71.4	71.4	100.0
	Missing	0	0		
Livelihood versus observed temperature changes					
	1: Crop farming				
	0: Rising temperature	188	87.9	87.9	87.9
	1: Declining temperature	13	6.1	6.1	94.0
	2: No change	13	6.1	6.1	100.0
	3: Missing	0	0		
	1: Livestock keeping				
	0: Rising temperature	4	57.1	57.1	57.1
	1: Declining temperature	0	0	0	57.1
	2: No change	3	42.9	42.9	100.0
	3: Missing	0	0		
	2: Employed				
	0: Rising temperature	63	86.3	86.3	86.3
	1: Declining temperature	2	2.7	2.7	89.0
	2: No change	8	11.0	11.0	100.0
	3: Missing	0	0		
	3: Trade				
	0: Rising temperature	18	85.7	85.7	85.7
	1: Declining temperature	1	4.8	4.8	90.5
	2: No change	2	9.5	9.5	100.0
	4: Fishing				
	0: Rising temperature	14	100.0	100.0	100.0
	1: Declining temperature	0	0	0	
	2: No change	0	0	0	
	3: Missing	0	0		
	5: Brick making				
	0: Rising temperature	0	0	0	0
	1: Declining temperature	0	0	0	0
	2: No change	1	100.0	100.0	100.0
	3: Missing	0	0		
Livelihood versus observed drought changes					
	1: Crop farming				
	0: Rising temperature	150	70.1	70.1	70.1
	1: Declining temperature	56	26.2	26.2	96.3
	2: No change	8	3.7	3.7	100.0
	3: Missing	0	0		
	2: Livestock keeping				
	0: Rising temperature	4	57.1	57.1	57.1
	1: Declining temperature	3	42.9	42.9	100.0
	2: No change	0	0	0	
	3: Missing	0	0		
	3: Employed				
	0: Rising temperature	60	82.2	82.2	82.2
	1: Declining temperature	12	16.4	16.4	98.6
	2: No change	1	1.4	1.4	100.0
	3: Missing	0	0		
	4: Trade				
	0: Rising temperature	19	90.5	90.5	90.5
	1: Declining temperature	2	9.5	9.5	9.5
	2: No change	0	0	0	100.0
	3: Missing	0	0		
	5: Fishing				
	0: Rising temperature	14	100.0	100.0	100.0
	1: Declining temperature	0	0	0	
	2: No change	0	0	0	
	3: Missing	0	0		
	6: Brick making				
	0: Rising temperature	0	0	0	0
	1: Declining temperature	1	100.0	100.0	100.0
	2: No change	0	0	0	
	3: Missing	0	0		
Livelihood versus observed rainfall changes					
	0: Crop farming				
	0: Declining rains	110	51.4	51.4	51.4
	1: Late rains	36	16.8	16.8	68.2
	2: Rains coming early	1	0.5	0.5	68.7
	3: Shorter rain seasons	31	14.5	14.5	83.2
	4: Longer rain seasons	22	10.3	10.3	93.5
	5: More rains	3	1.4	1.4	94.9
	6: No change	1	0.5	0.5	95.4
	7: Other	10	4.6	4.6	100.0
	Missing	0	0		
	1: Livestock keeping				
	0: Declining rains	3	42.9	42.9	42.9
	1: Late rains	1	14.3	14.3	57.2
	2: Rains coming early	0	0	0	57.2
	3: Shorter rain seasons	3	42.9	42.9	100.0
	4: Longer rain seasons	0	0	0	
	5: More rains	0	0	0	
	6: No change	0	0	0	
	7: Other	0	0	0	
	Missing	0	0		
Livelihood vs observed flood occurrence					
	0: Crop farming				
	0: Yes	46	21.5	21.5	21.5
	1: No	167	78.0	78.0	100.0
	Missing	1	0.5		
	1: Livestock keeping				
	0: Yes	6	85.7	85.7	85.7
	1: No	1	14.3	14.3	100.0
	Missing	0	0		
	2: Employed				
	0: Yes	41	56.2	56.2	56.2
	1: No	32	43.8	43.8	100.0
	Missing	0	0		
	3: Trade				
	0: Yes	17	81.0	81.0	81.0
	1: No	4	19.0	19.0	100.0
	Missing	0	0		
	4: Fishing				
	0: Yes	14	100.0	100	100.0
	1: No	0	0	0	
	Missing	0	0	0	
	5: Brick making				
	0: Yes	0	0.0	0	0
	1: No	1	100.0	100	100.0
	Missing	0	0		

With regards to gender of the household heads, the proportion of males and females in the study area were nearly equal with males accounting for 48.8 % and females 51.2 % of the respondents. This finding is not significantly different from that of the 2009 census report of the Kenya Bureau of Statistics (GoK 2010c) in which the distribution of males and females across the age groups were almost symmetrical. About 52.4 % of the respondents (household heads) had attained primary level of education, while 36 % had seconadry level of education. However, 4.2 % of the respondents had no education at all while only 4.8 and 2.1 % had attained tertiary and university education, respectively. These findings were nearly consistent with those reported in the KNBS 2009 census report which revealed that majority of Kenyans had attained primary level of education (51 %) followed by those who had attained secondary level of education at 17 % (GoK 2010c). However, while this study suggests that households are aware of the general change in climate, it established that there were varying levels of awareness of individual climate change markers (temperature, drought, heavy rainfall and floods among others).

Most (64.8 %) households interviewed practiced crop farming as the main source of livelihood. Other livelihood sources included: salaried employment (24.1 %), trade (6.4 %), fishing (4.2 %), livestock rearing (2.1 %) and brick making (0.3 %). The findings showed a general over-reliance on crop farming by a majority of households though few households had diversified their sources of livelihoods. This finding is not significantly different from that of Gichere et al. (2013) in which it was reported that farming was generally the main occupation for 63.2 % of households in the Lake Victoria Basin and SEI (2009) report which stated that 75 % of the Kenyan population base their livelihoods on agriculture.

### Level of climate change awareness

The results show that 90.9 % of the respondents reported having observed changes in the overall climate in their region while 9.1 % reported not having noticed any changes in climate in the region. Going by the responses, it is clear that the level of awareness of climate change is relatively high among residents of Upper Nyakach Division. Consistent with the current findings, a study on vulnerability assessment to climate change of the LVB inhabitants by the LVBC (2011) reported that most people in the Lake Victoria Basin had experienced climate change markers related to adverse changes in weather, while a study by Ndambiri et al. (2012) reported that 94 % of farmers in Kyuso District, Kenya, were aware of climate change and its effects. The current study findings also concur with those of Kabubo-Mariara and Karanja (2007) that most Kenyans are aware of short-term changes in climate.

Consistent with the current study findings, other studies done in and out of Africa such as that of Okonya et al. (2013) reported that nearly all households in agro-ecological zones of Uganda had observed climate change, while Deressa et al. (2008) found out that farmers in the Nile Basin of Ethiopia were highly aware of climate change. Oruonye (2011) also reported that 89.8 % of students of tertiary institutions in Jalingo Metropolis, Nigeria were aware of climate change, while Juana et al. (2013) concluded that climate change awareness is high based on an analysis of empirical studies conducted in Africa. However, all these studies including the current study contradict the earlier conclusions by Pelham (2009); GoK (2010a) and Mutimba et al. (2010) that climate change awareness levels amongst Kenyans is low.

**Fig. 1:**
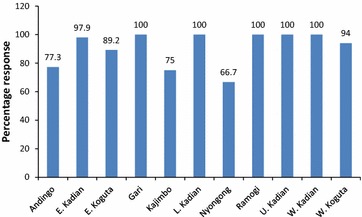
Awareness levels of climate change by sub locations

A Chi square test revealed that the level of awareness was significantly different across the 11 sub-locations (χ^2^ = 47.31, df = 10, P < 0.0001) despite their almost similar characteristics and proximity to each other. Indeed, the proportion of respondents who were aware of climate change in their area of residence was found to be lowest in Nyong’ong’a sub location as only 66.7 % reported observing changes in climate compared to other sub-locations. Considering the fact that Nyong’ong’a borders the shores of Lake Victoria and is prone to perennial flooding by River Sondu, the findings contradict the general expectation that it would post greater awareness than other sub-location. This could have been due to the inability of the respondents to link extreme weather events such as floods to climate change probably because of their proximity to the lake which is most often associated with a rise in water levels and not floods. Figure 1 shows the level of awareness on climate change across the 11 sub-locations.

Two regions that reported relatively lower level of awareness on climate change were Anding’o Opanga (77.3 %) and Kajimbo sub locations (75 %). However, in five sub-locations (Ramogi, West Kadianga, Upper Kadianga, Lower Kadianga and Gari), all (100 %) the respondents reported having observed some changes in climate in their respective areas. Further statistical analysis revealed that the difference in awareness of climate change across the age groups was not significant (χ^2^ = 4.317, df = 2, P = 0.116). Similar results were observed across gender (χ^2^ = 0.019, df = 1, P = 0.889) and level of education (χ^2^ = 3.901, df = 4, P = 0.420). However, it was observed that awareness of the households on changes in climate differed significantly across households’ main livelihood (χ^2^ = 18.458, df = 5, P = 0.002). This showed that the type of livelihood practiced by a household had some influence on climate change awareness with crop farmers and livestock keepers showing relatively higher level of awareness compared to traders, fishermen and brick makers.

### Observed changes in temperature

Most households (87 %) reported rising temperatures over the past 20 years while 4.8 % had observed declining temperatures. However, 8.2 % of the respondents had not observed any changes regarding temperatures in their area of residence. Going by the respondents’ observation on temperature changes in the division over the past 20 years, majority of the respondents were of the opinion that the temperature in their area has risen over that period. This is consistent with climate change projections by the IPCC that East Africa will experience warmer temperatures (Hulme et al. 2005; IPCC 2007). According to UNEP (2002) and Künzler (2011) the mean annual temperatures in Kenya had increased by 1 °C between 1960s and 2003 and the observations indicate significantly increasing trends in the frequency of hot days by 15.6 % per year, and much large increasing trends in the frequency of hot nights by 31 % per year. This has led to unpredictability of rains and drought patterns and the subsequent increase in vector borne diseases (Dida et al. 2015).

Likewise, SEI (2009) asserted that climate change projections indicated future increases in mean annual temperatures of between 1 and 3.5 °C by the year 2050 s. The current study findings concur with a number of studies done within and out of Africa. For instance, Ndambiri et al. (2012) reported high awareness of rise in temperatures among farmers in Kyusu District, Kenya. Likewise, most (87.5 %) respondents in a study conducted among crop farmers by Olayemi (2012) on the most noticeable climate change in Ondo state, Nigeria singled out high temperatures. Deressa et al. (2008) also reported that most framers in the Nile Basin of Ethiopia were aware that temperatures are rising. However, the proportion of respondents who reported having observed a rise in temperature in the current study was much higher (39 %) than that previously reported by Okonya et al. (2013) among farmers in agro-ecological zones of Uganda.

Based on the total sample, significant differences were observed across the age groups on the level of awareness of changes in temperature (χ^2^ = 25.316, df = 4, *P* < 0.001) with the middle age group (36–60 years) having observed more of a rise in temperature or no change in temperature while the respondents in the old age (above 60 years) category reported having observed more of a decline in temperature than other age groups. Similarly, significant differences in awareness of changes regarding temperature were observed across the levels of education of the respondents (χ^2^ = 41.708, df = 7, P = 0.011) with a higher proportion of respondents who had attained primary level of education having observed all the changes compared to other levels of education. The relatively high level of awareness on changes in temperature among respondents who had attained primary level of education as compared to other levels of education could be explained by the fact that majority (64.8 %) of the respondents practiced crop farming as their main source of livelihood and were therefore more likely to notice any changes relating to temperature because it is likely to affect their livelihood source directly.

Also, significant differences on awareness of the changes relating to temperature were observed across respondents’ gender (χ^2^ = 8.380, df = 2, P = 0.015), with males being more aware of declining temperature than females while the females were more aware of rising temperature than their males counterparts. In addition, significant differences on awareness of the changes relating to temperatures were also observed across the households’ main livelihood (χ^2^ = 12.631, df = 10, *P* = 0.002) with crop farmers being more aware of temperature changes compared to the respondents pursuing other livelihood types.

Based on the current study findings, age and gender of respondents had some influence on the level of awareness of changes observed in general climate change. This is consistent with the previous findings by Ndambiri et al. (2012) and Deressa et al. (2008) both of which reported that age, gender and education level had significant effects on respondent’s awareness of climate change.

### Observed changes in rainfall patterns

**Fig. 2:**
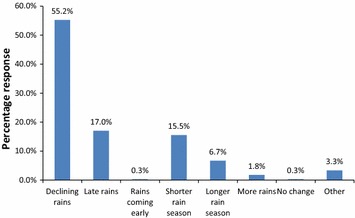
Observed changes in rainfall pattern

There have been changes asssociated with rainfall pattern in the area as 55.2 % of the respondents had observed declining rains, while 17.0 and 15.5 % had observed late rains and shorter rain seasons, respectively, while 6.7 % reported longer rain seasons and 1.8 % of the respondents observed more rains. Only 0.3 % of the respondents did not observe any change in the rainfall patterns. Figure 2 shows observed changes in rainfall patterns by proportion of respondents.

The findings shown on Fig. 2 indicate that over the past there have been changes in frequency and intensity of rainfall, with decline in rains being the most notable. Similar findings were reported by Ndambiri et al. (2012) in Kyuso District, Kenya where most farmers were reported to be aware of declining precipitation amounts. The current study findings also concurs with reports by NEMA (2013), GoK (2010a) and Künzler (2011) that there is a general decline in the long rainy season (March–April-May) over the past years in the country. Adaptation to Climate Change and Insurance (ACCI) commissioned a pilot project in Homa Bay and Busia Counties in Kenya, which revealed general declining trends in rainfall in both counties (www.acci.co.ke/accio/wp/climate-change/); the findings of which this study corroborates. However, the results of the IPCC (2007) prediction indicated that Eastern Africa will likely experience a modest (5–10 %) increase in June, July and August precipitation.

Significant differences were observed across the age groups on awareness of changes relating to rainfall patterns (χ^2^ = 41.396, df = 14, *P* < 0.001) with more respondents in the middle age group (36–60 years) being aware of the changes in rainfall patterns than the youth (30–35 years) and old age (above 60 years). Similar results were observed across gender as more males were aware of declining and late rains than females (χ^2^ = 41.708, df = 7, *P* < 0.001), while more female were aware of shorter rain seasons than their male counterparts. The fact that more male tended to identify late rains while more female tended to identify shorter rain seasons may give a glimpse into gender perspective of climate change awareness. Whereas males often involve themselves more with farm preparation and planting, they tended to be more aware of change in onset of rains while women who are often more involved in tending to crops (e.g. weeding) were more observant of the change in length of seasons. Also, significant differences on awareness of the changes in rainfall patterns were observed across the levels of education of respondents (χ^2^ = 71.315, df = 28, *P* < 0.001) with all the changes observed by respondents who had primary education as opposed to those with university education who only observed declining rains and late rains. As was the case for temperature, most respondents who had attained primary level of education and most of whom were also farmers were likely to observe declining rains as well as late rains. The current study finding is consistent with studies by Brulle et al. (2012) and Hasan and Akhter (2011), who reported that those with lower levels of education are likely to perceive climate change as a threat since they rely more on rain fed agriculture and have less income thus remain highly vulnerable to the impacts of climate change.

Similarly, awareness level of rainfall changes by respondents was significantly different across the livelihood types (χ^2^ = 126.910, df = 35, P < 0.001) with crop farmers being more aware of the changes in rainfall patterns. Again this is because crop farmers were likely to feel the impact of any fluctuations in rainfall patterns first owing to their livelihood source which depends wholly on rainfall.

### Observed changes in drought patterns

According to United States Geological Survey, USGS (2011), increased frequency of droughts observed in the last 20 years is likely to continue (as long as global temperatures continue to rise). This is evidenced by the current study findings as 75 % of the respondents reported that droughts had become more frequent over the years as opposed to 22 % who believed otherwise and 3 % who had not observed any change at all over the past 20 years. On the severity of drought, 68.5 % of the households had observed that drought events in the area are increasingly getting severe as opposed to 29.1 % who observed a decrease in severity of drought events. A small proportion (2.4 %) did not observe any change over the years.

The large proportion of households that had observed drought occurrence in the Upper Nyakach Division was consistent with findings of LVBC (2011) that drought events have increased within the Lake Victoria Basin (of which the study area is part). Majority of the households observed increased frequency (74 %) and severity (68.5 %) of drought events over the last 20 years, which is in agreement with the NEMA (2008) report in which serious drought events were reported to have occurred in the country at least 12 times in the past (50 years). Humanitarian Information Unit, HIU (2007) asserted that Kenya is frequently affected by severe drought and that based on historical rainfall records, there is greater than 40 % likelihood that Western Kenya will most likely experience severe drought within the rainy seasons of any given year. Studies by FAOSTAT (2000) and UNEP (2002) reported that the frequency and severity of droughts and floods have increased over the past 50 years, especially in Eastern Africa. This shows that the study finding on the trend of droughts in the area is consistent with both observed and projected drought trends at both national and regional levels.

A Chi square test revealed significant differences across gender of the respondents with regard to observed changes in drought frequency (χ^2^ = 10.592, df = 2, P = 0.005) with fewer male respondents having agreed that droughts are either getting less frequent or that there has been no change at all compared to their female counter parts. However, the differences regarding observed changes in drought frequency were not significant among the age groups (χ^2^ = 6.148, df = 4, P = 0.188) as was with the levels of education (χ^2^ = 25.131, df = 8, P = 0.291) and households’ livelihoods type (χ^2^ = 22.490, df = 10, P = 0.060). These findings contradicts those of Deressa et al. (2008) and Ndambiri et al. (2012) which reported that gender and education level of respondents had significant effects on awareness of climate change.

### Observed changes in flood occurrence

Awareness of flood occurrence was assessed on the basis of whether the respondents had observed floods phenomena in the area over the last 20 years. The study findings revealed that 37.6 % of the households reported having observed flood occurrence in Upper Nyakach Division while 62.4 % had not observed occurrence of floods. This implied that only a small proportion of the households were aware of flooding in parts of the study area over the past. These findings were almost similar to those cited in a report on vulnerability assessment by the LVBC (2011) that 35.9 % of households in the Lake Victoria Basin had experienced floods. According to the State of Environment Report of 2006/2007 (NEMA 2008), major floods periodically afflict Lake Victoria Basin, the Lower Tana Basin and the coastal region—occurring at least six times in the past 50 years. This has been corroborated by the current study findings, which are also in agreement with the study by Okonya et al. (2013) which reported that floods have increased in the agro-ecological zones of Uganda over the past 10 years.

There was significant difference across the 11 sub-locations regarding awareness of flood occurrence (χ^2^ = 213.524, df = 70, P > 0.0001) with greater proportion of respondents from lowland sub-locations (Lower Kadianga, Upper Kadianga, West Kadianga, West Koguta and Nyong’ong’a) having observed or experienced flooding events over the past 20 years. This finding is consistent with Thodsen et al. (2014) who reported an increase in flood levels and frequencies in lowlands in Denmark.

### Observed changes on water sources

**Fig. 3:**
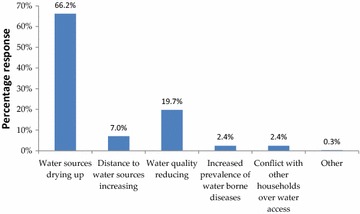
Major change observed on water sources

Majority of the households (86.7 %) reported having observed changes in water sources compared to 13.3 % who did not observe any changes. The actual changes observed were drying up of water sources (63.3 %), reduction in water quality (17.3 %), conflicts over water access (12.7 %), increasing distance to water sources (5.8 %) and a rise in the prevalence of water borne diseases (0.9 %) as shown in Fig. 3.

**Fig. 4:**
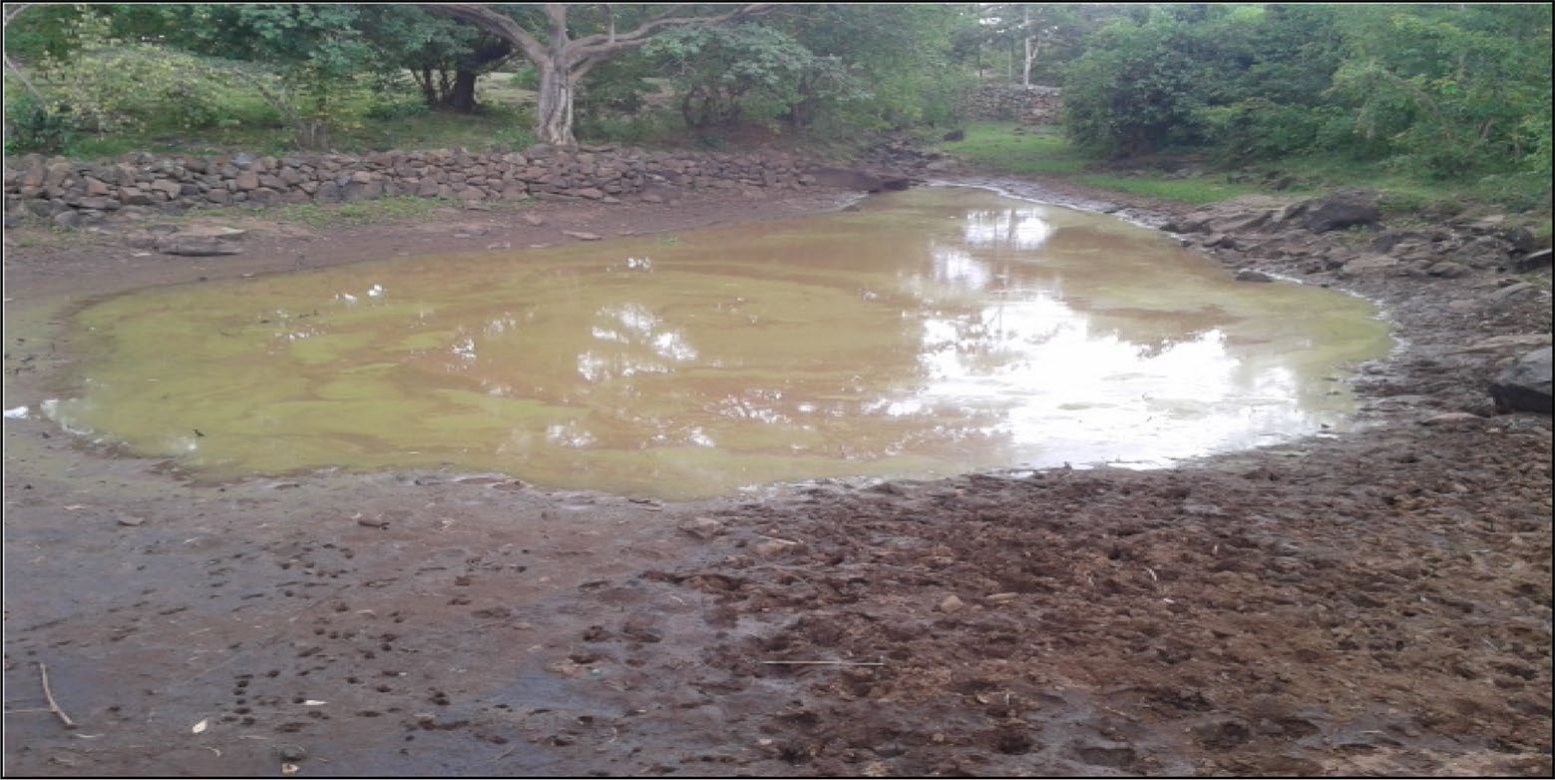
A shrinking community water dam used for watering livestock and domestic needs in East Koguta (*Source*: Field data)

Indeed, some springs were observed to be drying up and their water quality also reducing. This acted as further evidence on the reported drying of water sources in the area. As a result of reduction of water quality and/or drying up, some springs have been abandoned by households. Figure 4 shows a spring at the foot of Nyabondo hill that was abandoned due to its reduced water quality.

The current study finding on observed changes in water sources are consistent with LVBC (2011) report which showed that distance to the water sources is increasing for the households in the Lake Victoria Basin (of which the study area is part) and some water sources have been drying up. Also, according to NEMA (2008), Mutimba et al. (2010) and GoK (2010a) major droughts experienced in the country over the past 50 years have seen severe reduction in the volume of water across the country’s major rivers while the seasonal ones have completely dried up.

A Chi square test revealed significant differences across the age groups relating to awareness of the changes in water sources (χ^2^ = 31.92, df = 10, P > 0.0001) with the respondents in the middle age being more aware of the changes than the youth and the elderly. This could have been largely due to the fact that the respondents were concentrated around the middle age group. Similar results on the awareness on changes relating to water sources were observed across the livelihood types (χ^2^ = 59.020, df = 25, P > 0.0001) in which livestock farmers and fishermen were most aware of changes in water sources. This could be due to the fact that their livelihoods revolve around the water sources, are therefore more likely to notice any slight changes in water sources. However, no significant difference was observed on awareness on the changes relating to water sources across gender (χ^2^ = 6.04, df = 5, P = 0.302) as was the case across levels of education (χ^2^ = 27.678, df = 20, P = 0.117). These findings however contradicted those of Deressa et al. (2008) and Ndambiri et al. (2012) in which age, gender and education were reported to have significant effect on climate change awareness.

### Observed changes in malaria prevalence

**Fig. 5:**
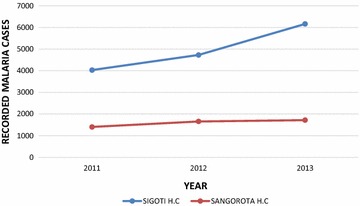
Trend of malaria cases recorded at Sigoti and Sango Rota health centres between 2011 and 2013

Majority of the respondents interviewed (74 %) reported an increased prevalence of malaria. However 23 % of the households believed that fewer people were falling ill (malaria cases have reduced) while 3 % have seen no change at all regarding malaria cases in their area over the past 20 years. These findings seem to suggest that malaria infections have increased over the past, a fact that was confirmed by data from two selected health centres in the study area (Sigoti and Sango Rota) between 2011 and 2013. Figure 5 shows the trend of malaria cases recorded in Sigoti and Sango Rota Health Centres between 2011 and 2013.

This finding is consistent with the observation by the National Climate Change Response Strategy (NCCRS) that one of the potential impacts of climate change will be an increase in the incidence and geographical spread of vector borne diseases such as malaria (GoK 2007) and the finding that climate change could increase the rural population at risk of malaria by 2050s (SEI 2009). It also confirms the report by LVBC (2011) which found malaria to be the most notorious disease within the Lake Victoria Basin.

Significant differences were observed among the different education levels on awareness of changes relating to malaria cases (χ^2^ = 19.369, df = 8, P = 0.013) with respondents who attained primary and secondary level of education and above having observed increasing cases of malaria (by proportion). Similar results were observed across the households’ main livelihoods (χ^2^ = 14.331, df = 10, P < 0.0001) with the proportion of those who had observed an increase in malaria cases over the past years being larger amongst fishermen and livestock keepers than amongst those engaged in other forms of livelihoods. These findings contradicted those of the LVBC (2011) which stated that differences in climate change awareness among respondents with different livelihoods (occupation) were not significant. In the current study, differences observed on awareness of the changes in malaria cases among different age groups were not significant (χ^2^ = 4.607, df = 4, P = 0.330). The current findings concurred with those of Deressa et al. (2008) and Ndambiri et al. (2012) which also reported that gender and age have significant effect on climate change awareness. This gender gap could be due to differences between men and women in access to weather information, education differences, or lack of basic channels of communication such as radio and television set.

### Regression model of determinants of climate change markers awareness

Overall, from the full Poison–GLm model of important variables for the awareness of climate change markers (Table 2), only gender of the household head (when the gender was a male), education and age of the respondents significantly predicted awareness of climate change markers (Table 3) even as youth and middle age (borderline significant) groups were found to be more aware of the climate change markers than their adult counterparts.

**Table 2:** Full Poisson-GLM model for estimate, standard errors (SE), Z-value and p-values for important variables for the awareness of climate change markers

Variable	Estimate	SE	Z value	Pr(> |z|)
Intercept	−0.30168	0.09127	−3.305	0.000949***
Male	0.29812	0.04594	6.489	8.64e−11***
Female	0.06156	0.05744	0.305	0.317230
Uneducated	0.03233	0.07601	0.425	0.670576
Primary school level	0.17164	0.09262	4.777	0.005492**
Secondary school level	0.18696	0.08678	3.462	0.008148**
Tertiary level	−0.02955	0.08691	−0.342	0.732265
Old age	0.05046	0.07431	0.679	0.497094
Middle age	−0.07177	0.05103	−2.406	0.059581*
Youth	0.14126	0.04844	3.216	0.003543**

**Table 3:** Final Poisson GLM model for estimate, standard error (SE), Z-value and P-values of important variables for the awareness of climate change markers

Variable	Estimate	SE	z-value	Pr(>|z|)
Intercept	−0.30	0.09	−3.73	<0.001***
Male	0.32	0.05	5.81	<0.001***
Primary school level	0.41	0.06	6.32	<0.001***
Secondary school level	0.07	0.08	4.79	0.001**
Youth	0.14	0.05	4.44	0.003**

* P < 0.05; *** P < 0.001

The current findings are inconsistent with those reported by LVBC (2011) which previously found insignificant differences between climate change awareness with background factors. However, the study support findings by Ndambiri et al. (2012) which showed a relationship between age, gender and education and climate change awareness. Similarly, Deressa et al. (2008) reported significant differences in climate change awareness with respect to education level and age although their study looked at age in general. Although education level of the respondent was found to have an impact on the awareness of the respondent in the current study, the influence was not significant at tertiary level of education. This could be due to the fact that few individuals in this category might have specialized in other areas of interest and had little interest on matters of climate change.

## Conclusions

The current study findings show that the level of awareness of climate change in the Upper Nyakach Division is relatively high as most households in the area reported having observed overall changes in climate. Climate change awareness varied significantly across different sub-locations. This makes Upper Nyakach Division  a potential area for further insight analysis, because such sub- geographical location differences affects households and their livelihood sources. The study findings revealed that demographic factors such as age and gender of the household head have significant influence on the general awareness on climate change.

## Additional file

10.1186/s40064-016-2699-y A questionnaire to determine the level of awareness of climate change markers was divided into two broad sections, namely: (1) Household characteristics and (2) Awareness of climate change markers. The 1st section addressed age, gender, education level, marital status, main livelihood and sources of food while the second section addressed the changes observed in different climate change markers (temperature, rainfall, drought, floods, water availability and prevalence of malaria) over the last 20 years.

## Abbreviations

ACCI: Adaptation to Climate Change and Insurance
AMCEN: African Ministerial Conference on Environment
FAOSTAT: Food and Agriculture Organization Statistics
FGDs: Focus Group Discussions
GDP: Gross Domestic Product
HIU: Humanitarian Information Unit
IPCC: Inter-governmental Panel on Climate Change
KII: Key Informant Interviews
LVB: Lake Victoria Basin
LVBC: Lake Victoria Basin Commission
NCCRS: National Climate Change Response Strategy
NEMA: National Environment Management Authority
SAS: Statistical Analysis Software
SEI: Stockholm Environment Institute
SPSS: Statistical Package for Social Science
UNFCCC: United Nations Framework Convention on Climate Change
USGS: United States Geological Survey
