# Supplementary material for: Determinants of climate change awareness level in upper Nyakach Division, Kisumu County, Kenya
Source: Springerplus. 2016 Jul 8;5(1):1015. doi: 10.1186/s40064-016-2699-y (PMC4938833; doi:10.1186/s40064-016-2699-y)
Supplement: Supplementary file 1 — 10.1186/s40064-016-2699-y A questionnaire to determine the level of awareness of climate change markers was divided into two broad sections, namely: (1) Household characteristics and (2) Awareness of climate change markers. The 1st section addressed age, gender, education level, marital status, main livelihood and sources of food while the second section addressed the changes observed in different climate change markers (temperature, rainfall, drought, floods, water availability and prevalence of malaria) over the last 20 years. [file 40064_2016_2699_MOESM1_ESM.docx]

|  |
| --- |

**Questionnaire on awareness level of climate change markers administered on 364 respondents of Upper Nyakach Division, Kenya**

| **SECTION 1: HOUSEHOLD CHARACTERISTICS** | | | |
| --- | --- | --- | --- |
| **Question No.** | **Question** | **Response** | **Response Code** |
| 1.1 | What is your age? (***years completed***) | ………………………………………..Yrs | |
| 1.2 | Gender of the respondent | 1 = Male  2 = Female |  |
| 1.3 | What is your highest level of education attained? | 1 = None  2 = Primary  3 = Secondary  4 = Tertiary  5 = University |  |
| 1.4 | What is your marital status? | 1 = Married  2 = Single  3 = Widow/ Widower  4 = Separated/ Divorced |  |
| 1.5 | If married, what is the highest level of education attained by spouse? | 1 = None  2 = Primary  3 = Secondary  4 = Tertiary  5 = University |  |
| 1.6 | Type of household | 1 = Male Headed  2 = Female Headed  3 = Youth Headed |  |
| 1.7 | What is your **MAIN** livelihood? | 1 = Crop Farming  2 = Livestock Keeping  3 = Casual Employment  4 = Salaried Employment  5 = Trade  6 = Fishing  7 = Brick Making  9 = Others (Specify)……………………………… |  |
| 1.8 | What is your household’s **MAIN** source of food | 1 = Crop Grown On Farm  2 = Animal Produce From Animals Reared  3 = Buy From The Market  4 = Fish From The Lake  5 = Donations From Relatives  6 = Relief From Agencies  9 = Other (Specify)…………………………… |  |
| **SECTION 2: AWARENESS ON CLIMATE CHANGE MARKERS** | | | |
| **Question No.** | **Question** | **Response** | **Response Code** |
| 2.1 | Have you noticed change in the climate of this area over the last 20 years? | 1 = Yes  2 = No |  |
| 2.2 | If yes, through which markers/ indicators did your notice this change? (***multiple choices allowed)*** | 1 = Changing temperatures  2 = Change in rain patterns  3 = Change in drought patterns  4 = Change in flood patterns  5 = Change in water availability  6 = Increased prevalence of malaria |  |
| 2.3 | What is the **MOST SIGNIFICANT** maker through which you noticed the change in climate of the area? | 1 = Rising temperatures  2 = Change in rain patterns  3 = Change in drought patterns  4 = Change in flood patterns  5 = Change in water availability  6 = Increased prevalence of malaria |  |
| 2.4 | What kind of change have you noticed regarding night and day temperatures of this area? | 1 = Rising temperatures  2 = Declining temperatures  3 = No change |  |
| 2.5 | What kind of change have you noticed in the rainfall patterns of the area? | 1 = Declining rains  2 = Late rains  3 = Rains coming early  4 = Shorter rain seasons  5 = Longer rain seasons  6 = More rains |  |
| 2.6 | How frequent has the frequency of drought events grown over the last 20 years? | 1 = More frequent  2 = Less frequent  3 = No change |  |
| 2.7 | What trend in severity of the effects of drought have you noticed over the last 20 years? | 1 = Increasing severity  2 = Decreasing severity  3 = No change |  |
| 2.8 | Has your area been affected by floods | 1 = Yes  2 = No |  |
| 2.9 | If yes, how frequent have flood events grown over the last 20 years | 1 = More frequent  2 = Less frequent  3 = No change |  |
| 2.10 | What trend in severity of the effects of drought have you noticed over the last 20 years? | 1 = Increasing severity  2 = Decreasing severity  3 = No change |  |
| 2.11 | Have you noticed any change in water sources in your area? | 1 = Yes  2 = No |  |
| 2.12 | If yes, through which indicators did you notice this? (***multiple responses allowed)*** | 1 = water sources drying up  2 = distance to water sources increasing  3 = water quality reducing  4 = increased prevalence of water-borne diseases  5 = Conflict with other households over water access  9 = Other (specify) ………………… |  |
| 2.13 | What changes change has occurred in your area related to malaria infections over the last 20 years | 1 = More people are falling ill  2 = Few people are falling ill  3 = No change |  |

**THAT WAS THE LAST QUESTION. THANK YOU!!**
